# Homology-directed gene-editing approaches for hematopoietic stem and progenitor cell gene therapy

**DOI:** 10.1186/s13287-021-02565-6

**Published:** 2021-09-09

**Authors:** Manoj Kumar K. Azhagiri, Prathibha Babu, Vigneshwaran Venkatesan, Saravanabhavan Thangavel

**Affiliations:** 1 Centre for Stem Cell Research (CSCR), a Unit of InStem Bengaluru, Christian Medical College Campus, Vellore, Tamil Nadu India; 2 Manipal Academy of Higher Education, Manipal, Karnataka India

**Keywords:** Hematopoietic stem and progenitor cells, Gene-editing and homology-directed repair

## Abstract

The advent of next-generation genome engineering tools like CRISPR-Cas9 has transformed the field of gene therapy, rendering targeted treatment for several incurable diseases. Hematopoietic stem and progenitor cells (HSPCs) continue to be the ideal target cells for gene manipulation due to their long-term repopulation potential. Among the gene manipulation strategies such as lentiviral gene augmentation, non-homologous end joining (NHEJ)-mediated gene editing, base editing and prime editing, only the homology-directed repair (HDR)-mediated gene editing provides the option of inserting a large transgene under its endogenous promoter or any desired locus. In addition, HDR-mediated gene editing can be applied for the gene knock-out, correction of point mutations and introduction of beneficial mutations. HSPC gene therapy studies involving lentiviral vectors and NHEJ-based gene-editing studies have exhibited substantial clinical progress. However, studies involving HDR-mediated HSPC gene editing have not yet progressed to the clinical testing. This suggests the existence of unique challenges in exploiting HDR pathway for HSPC gene therapy. Our review summarizes the mechanism, recent progresses, challenges, and the scope of HDR-based gene editing for the HSPC gene therapy.

## Introduction

Recent estimates suggest that over 8000 diseases are of monogenic in origin, often manifesting during childhood and causing premature deaths in severe cases. The burden of genetic disorders remains alarmingly high as the clinical management of many such diseases is largely inefficient. For monogenic disorders, such as β-hemoglobinopathies (β-thalassemia and sickle cell disease (SCD)), cystic fibrosis, hemophilia, Huntington’s disease and Duchenne muscular dystrophy, targeted therapeutic strategies are in high demand.

Gene therapy aims to correct the root cause of monogeneic disorders by directly acting at DNA level and by employing a wide array of viral or nuclease-based strategies such as gene supplementation, silencing, correction or disruption. Gene augmentation using viral vectors is the most clinically advanced strategy; however, this has lately been known to posit risks such as random integration, insertional mutagenesis and immunogenicity [1]. The development of customizable DNA cleaving endonucleases such as meganucleases, zinc finger nucleases (ZFNs), transcription activator-like effector nucleases (TALENs) and clustered regularly interspaced short palindromic repeats-associated RNA-guided Cas9 (CRISPR-Cas9) revolutionized the field of gene editing and allowed facile gene manipulation.

Nuclease-mediated gene editing is achieved by exploiting the intrinsic DNA repair pathways such as non-homologous end-joining (NHEJ) and homology-directed repair (HDR) which are activated following the generation of double-stranded breaks (DSB). The NHEJ repair results in the direct ligation of the cleaved strands producing InDels (insertions–deletions) and is predominantly used for gene disruption, whereas HDR follows a directed correction strategy where an exogenous repair template with the desired nucleotide sequence mediates the process. Thus, the targeted genome-editing strategies hold a great promise for establishing precision edits and can overcome the risks associated with the viral-based strategies [1, 2]. A comparison between gene addition and genome-editing strategies is listed in Table 1.

**Table 1:** Comparison of gene addition and genome-editing strategies

	viral-mediated gene addition strategies	HDR-based genome-editing strategies	NHEJ-based genome-editing strategies
Therapeutic gene expression	Depending on the vector copy number and the integration sites	Physiological level expression from endogenous locus Supraphysiological levels from safe harbor, alpha globin locus	–
Efficiency in human HSPCs	Depends on transduction efficiency Transgene, promoter and other regulatory sequences in the vector	Depends on frequency of HDR events, donor types, donor sequences & availability at cut site	Frequency of productive indels
Genotoxic risks	Oncogene transactivation, generation of aberrant transcripts, gene inactivation	Indels at HDR site Off-targets, large genomic rearrangements	Off-targets, large genomic rearrangements
Costs	High (viral delivery)	High (viral delivery)/low (nonviral delivery)	Low (nonviral delivery)

HSPCs are the ideal targets for the gene therapy of many hematological and immunological disorders. Using HSPCs for gene therapy provides potential long-term benefits as they replenish the patient’s hematopoietic system with gene-modified stem cells. Establishing efficient HDR editing in HSPCs is crucial for attaining favorable therapeutic outcomes [3]. This review discusses the applications of HDR in HSPC gene therapy, challenges and the possible solutions.

## Homology-directed repair of DNA breaks

**Fig. 1:**
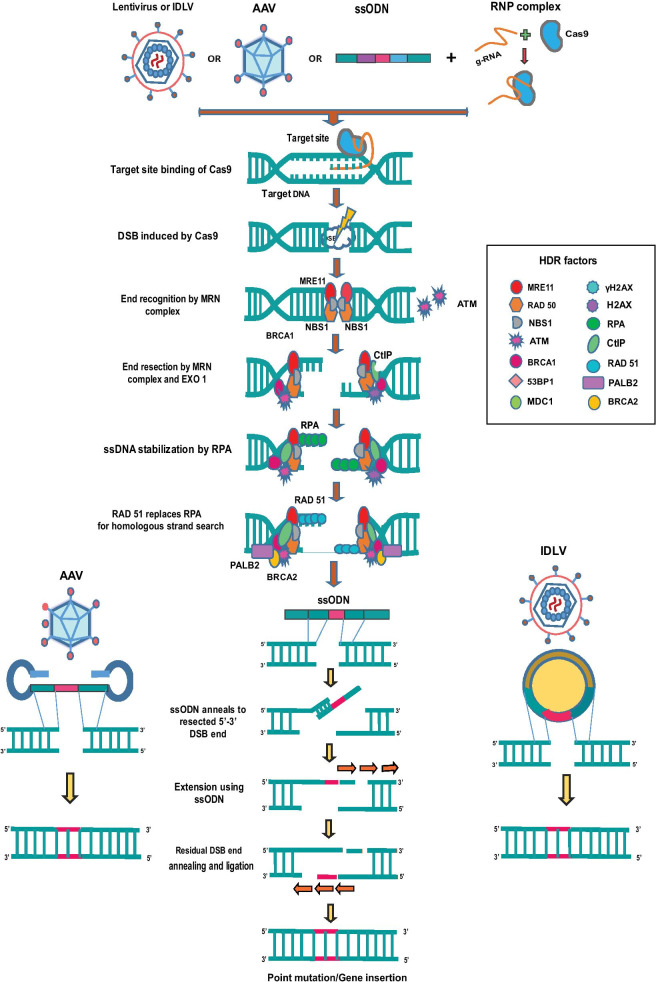
HDR gene-editing. Cas9-RNP along with a donor template (AAV6 or ssODN or IDLV) is delivered into HSPCs. Cas9 RNP introduces DNA double-stranded breaks at the target locus and endogenous HDR pathway repairs the DNA damage. During this process, the donor template having homologous sequence to the cut site is inserted into the target locus

The cell cycle checkpoints and intrinsic DNA repair pathways help to curb the detrimental genomic damages exerted by various genotoxic agents, exogenous nucleases and replication stress. Most genomic lesions are corrected either by NHEJ or HDR pathways, the choice of which is dependent on the DNA resection at DSB site as well as recruitment of repair proteins. The HDR pathway is illustrated in Fig. 1.

53BP1 and BRCA1 are the two key factors that regulates DNA resection. 53BP1 antagonizes the DNA end resection by forming a complex with RIF1 which blocks the BRCA1 recruitment to the DNA DSBs. Thus, the unresected DSBs undergo NHEJ repair pathway which is active throughout interphase. The unresected DSBs also activate ATM checkpoint, coordinating DNA repair and cell cycle. When cells enter S-phase of the cell cycle, CDK-mediated S372 phosphorylation of CtIP activates CtIP and mediates the interaction with BRCA1. BRCA1-CtIP complex promotes dephosphorylation and repositioning of 53BP1 creating a chromatin environment for DNA end resection machinery. The BRCA1-CtIP forms a major complex with MRE11–RAD50–NBS1 (MRN) proteins, and this complex generates a short 3’overhanging single-stranded DNA at the DSB ends through the nuclease activity of MRE11. The generated ssDNA is immediately stabilized by replication protein A (RPA), and the short ssDNA is further extended by a helicases and nucleases complex which involves Bloom syndrome protein (BLM) and exonuclease 1 (EXO1) or WRN and DNA replication helicase/nuclease 2 (DNA2). The ssDNA also activates ATR cell cycle checkpoint. The end resection is terminated by BRCA2 mediated RAD51 loading which evicts RPA and forms nucleoprotein filaments with ssDNA. RAD51 nucleoprotein filaments promote homologous search and invasion of the repair template for the start of the repair process [4–6]

The complex events after the DSB vary with the type of HDR—canonical HDR, synthesis-dependent stranded annealing (SDSA), break-induced replication (BIR), single-stranded annealing (SSA) and single-stranded templated repair (SSTR). Although the major steps including end resection, homologous template search, synthesis of new strand from the template, ligation of the DNA ends run similar across all types of HDR, the subsequent steps vary with the type of HDR intended. In canonical HDR, 3’ overhang of the ssDNA invades the similar DNA duplex by strand invasion and D loop is formed between the 3’ overhang strand and the homologous DNA template. DNA polymerase extends the 3’ invading strand, and this changes the D loop to cross-shaped structure known as holliday junction. The resolution of double holliday junction results in recombination such as either crossover or non-crossover with the help of nicking endonucleases. In SDSA pathway, the resected ssDNA undergoes strand invasion into a homologous duplex strand forming a D-loop and primes the synthesis of nascent DNA. The nascent DNA is then dissociated to anneal with the other resected strand. In BIR, one end of the DSB end is available for strand invasion and other end does not engage with the homologous sequence. This results in asynchronous leading and lagging strand synthesis-mediated new DNA. SSA is initiated when DSB happens between two repeated sequences that are oriented in same direction. SSA requires long end resection of 200 bp and is independent of RAD51. RAD52 binds to the RPA coated ssDNA ends and anneals the resected single-stranded homologies to the DNA template. During the repair, single-stranded regions are made adjacent to the break which extends to the repeated sequences; thereby, complementary strands can anneal to each other. Finally, heterogenous flaps are removed by ERCC1-XPF nuclease and DNA gaps are ligated together. In SSTR pathway, the single-stranded DNA template gets annealed to the resected DNA strand and DNA extension is carried out with the help of DNA polymerase delta. The extended DNA product is now dissociated from the template, and the DNA ends are ligated using Ligase1 or its analogues [7, 8]. The resultant new strand of DNA is perfectly repaired using sequences from the template (Fig. 1).

## HDR gene editing

Gene editing relies on engineered nucleases to recognize and cut specific DNA sequences and subsequently exploits the innate DNA machinery of the cell to repair the nuclease-induced DSB. Supplementation of homologous DNA template with site specific nucleases shifts the innate DNA repair pathway to HDR attaining targeted nucleotide changes. However, only cells in the S/G2-phases have phosphorylated CtIP to negate the antagonizing effect of 53BP1 on BRCA1 and thus respond to HDR donor supplementation. All HDR donor templates contain left (5’) and right (3’) homology sequences flanking the cut site, to exploit the HDR pathway. A wide range of viral derived or synthetically generated double-stranded and single-stranded HDR donor templates are currently tested for single nucleotide changes to large transgene insertion. Viral-derived HDR donors include integrase defective lentiviral vectors (IDLVs), adenovirus 5/35 serotype (AdVs), and adeno-associated vector (AAV) and single-stranded oligo deoxynucleotides (ssODNs) is a non-viral option.

## IDLV-HDR donor

IDLVs were initially developed as alternatives to integrating viral vectors to avoid the risk associated with insertional mutagenesis. The D116 mutation within the catalytic domain of the integrase inactivates the integrase-mediated viral DNA integration and the resultant IDLV remains episomal which dissipates during cell divisions. This transient property makes IDLV a suitable HDR donor. IDLVs are free ended double-stranded DNA vectors, with the potential cargo delivery up to 10 kb [9]. IDLV templates have been tested in HSPCs for correcting SCID-X1 and SCD mutations. The results showed marginal levels of HDR (2–18%) with post-transplant reduction in HDR frequency (0.27–2%) [10–12]. The low frequency of target modification is due to the direct recombination of IDLVs with the target site before the initiation of HDR resulting in concatemer formation. In comparison with other HDR donors, IDLV donors exhibit reduced cytotoxic properties and it is presumed that the advancements in lentiviral transduction protocols such as cyclosporin H pre-treatment of HSPCs can be extended to IDLV donor templates for improving their efficiency [13].

## AdV HDR donor

AdVs are dsDNA vectors with protein-capped ends. Commonly used AdV vectors are derived from the serotype 5 modified to allow insertion of larger transgene (~ 35 kb). AdV exhibits broad range of tropism and high transduction efficiency [14]. Due to their large gene carrying capacity, they are used for delivery of the genes encoding the DNA cleaving endonucleases and the DNA templates for targeted insertion. The chimeric vector hybrid of serotypes 5 and 35 termed Ad5/35 has been used as HDR template, and it generates low efficiency HDR conversions (~ 2%) in cord blood (CB) HSPCs at HBB gene locus [12].

## AAV6 HDR donor

Ever since the discovery, AAV vector has been an important component for gene therapy. However, the existence of antibodies against AAV limits the application in in vivo gene therapy. AAV vectors are designed for the incorporation of moderately large DNA transgene (~ 5 kb). AAV6 serotype, which exhibits tropism toward HSPCs, is extensively used as donor delivery vector. AAV6 donor templates are designed with long homology arms of ~ 300–800 bp and result in high frequency of HDR in HSPCs and several other primary cell types [15]. AAV6 is reported to show successful target insertion of reporter constructs up to 91%, and AAV6 donors exhibit efficient HDR-mediated correction of sickle mutation than ssODN (50–60% vs 29.6%) in vitro, but the efficiency drops significantly in the long-term engrafted cells than the ssODN (32% vs 17.5% %) [12, 16–18].

## ssODN-HDR donor

ssODNs are short oligos often extending not more than 200 bp with homology arms in the range of 30–60 nucleotides flanking the desired nucleotide change. Compared to viral vectors, ssODNs possess several advantages such as simple design, reproducibility, short production time and relatively low cost. This makes them amenable to high-throughput applications. Asymmetric ssODNs (respective to the cut site) are efficient HDR templates than symmetric, and oligos complementary to non-target strand are highly effective as the Cas9 cleavage releases the 3’ end of non-target strand making it amenable to ssODN. Asymmetric ssODN (PAM proximal nucleotide 91 and PAM distal nucleotides 36 flanking genomic breakpoint) facilitates HDR rates up to 60% in 293-T cells [12, 15]. However, such inferences on the ssODN design determining the HDR efficiency are not uniform across different cell types, loci and donors. Targeted corrections were obtained in HSPCs, in a wide range of loci like HBB and IL2RG [19]. HBB gene editing with ZFN and ssODN co-delivery results in HDR frequency of 5–15%, and the efficiency decreased to 9% in the transplanted animals [12]. This indicates that optimized strategies must be developed for enhancing HDR rate by ssODN-based correction. ssODNs are the preferred substrates for SSTR, and the mechanism involved in ssODN-mediated repair is by direct annealing to the target DNA. ssODN approaches are beneficial for the correction of few nucleotides [15]. The major drawback of ssODN approach is that it cannot be applied for inserting a large transgene. Recently, Marson’s group overcame this limitation by using a long ssODN and achieved the targeted integration of > 1 kb CAR construct with an efficiency up to 12.3% in the TRAC locus of the T-cells [20]. Such approach is yet to be tested in HSPCs.

## HDR in HSPC gene therapy

Correction of defective gene by targeted gene manipulation is an attractive strategy in HSPC gene therapy. Editing of HSPCs provides long-term cure since these cells lie on the top of the hematopoietic hierarchy and effects can be retained in all the lineages, offering therapeutic benefits for both the hematological and immunological disorders. There are many studies showing correction of mutation using HDR-based gene editing in HSPCs for gene therapy application (Table 2).

**Table 2:** Therapeutic HDR gene-editing strategy for genetic disorders using ZFNs, TALENs and Cas9

Disease	Nuclease	Target locus	HDR-gene-editing strategy	Delivery route	Experimental model	References
Sickle cell disease & β-thalassemia	ZFNs	HBB	ZFN mRNA and IDLV/ssODN	Electroporation (Harvard apparatus)	HSPCs from healthy donor and SCD patient	[10]
	Cas9	HBB	Cas9 RNP and plasmid donor	Electroporation (Lonza Nucleofector 4-D)	HSPCs from healthy donor and SCD patient	[23]
			Cas9 mRNA and ssODN	Electroporation (Neon transfection system and Lonza 4D Nucleofector)	HSPCs from healthy donor	[24]
			Cas9 mRNA and IDLV	Electroporation (ECM 830 Square wave electroporator)	HSPCs from SCD patient	[53]
			Cas9 RNP and ssODN	Electroporation (Lonza 4D Nucleofector)	HSPCs from healthy donor	[22]
			Cas9 RNP and ssODN	Electroporation (Lonza 4D Nucleofector)	HSPCs from healthy donor	[54]
			Cas9 RNP and plasmid donor	Electroporation (Lonza Nucleofector 2b)	HSPCs from SCD patient	[55]
X-SCID	ZFNs	IL2RG	ZFN mRNA and IDLV	Electroporation (Lonza)	HSPCs from healthy donor and SCID-X1 patient	[57]
			ZFN and IDLV	Transduction	HSPCs from healthy donor	[58]
	Cas9		Cas9 RNP and AAV6	Electroporation (Lonza nucleofector 4D)	HSPCs from SCID-X1 patient	[30]
X-linked chronic granulomatous disease	Cas9	CYBB	Cas9 RNP and ssODN	Electroporation (MaxCyte systems)	HSPCs from X-CGD patient	[29]
Wiskott–Aldrich syndrome	Cas9	WAS	Cas9 RNP and AAV6	Electroporation/transduction (MaxCyte CTX Flow electroporator)	HSPCs from healthy donor and WAS patient	[17]
Mucopolysaccharidosis	Cas9	CCR5	Cas9 RNP and AAV6	Electroporation/transduction (Lonza nucleofector 4D)	HSPCs from healthy donor	[34]
Gaucher disease	Cas9	CCR5	Cas9 RNP and AAV6	Electroporation/transduction (Lonza 4D nucleofector)	HSPCs from healthy donor	[59]
Pyruvate Kinase Deficiency	TALEN& Cas9	PKLR	TALEN plasmid/Cas9 RNP and plasmid donor	Electroporation (Lonza AMXA II nucleofection system)	HSPCs from healthy donor	[27]
IPEX syndrome	Cas9	FOXP3	Cas9 RNP and AAV6	Nucleofection/transduction (Lonza 4D nucleofector)	HSPCs from healthy donor	[32]

### Blood disorders

β-Hemoglobinopathies: SCD and β-thalassemia are the defects associated with the production of β-globin chains qualitatively (SCD) and quantitatively (β-thalassemia) [21]. SCD arises due to a point mutation in the HBB exon (A > T or E6V), and mutation correction has been attempted with different types of HDR donor constructs. Nearly 50% of the E6V (HbS) alleles were reverted to wild type alleles by using RNP, AAV6 donor and selection of HDR cells. Similarly, 33% correction was achieved in a RNP and ssODN-mediated selection/enrichment free strategy and up to 23.4% of corrected alleles were retained for a long term in engrafted hematopoietic stem cells [22]. Another study demonstrated the correction efficiency of 24.5 ± 7.6% in vitro and 10% of corrected cells possessed long-term repopulation potential [23]. In line with this, targeted correction of β-thalassemia splicing variant IVS1-110 was achieved with Cas9 mRNA and ssODN with an efficiency of 8% [24]. Correction of wide range of β-thalassemia mutations requires personalized HDR-mediated gene correction approaches. As an alternative to mutation specific approach, a universal approach is developed which involves the reactivation of developmentally silenced γ-globin to compensate the decreased/defective β-globin chains. Several targets, such as − 175 T > C, − 195 T > C and − 113A > G HPFH mutations, are known to induce γ-globin re-activation and are demonstrated successfully in erythroid cell lines K562 and HUDEP-2. These targets are yet to be tested in HSPCs [25].

Hemophilia: Liver-targeted gene therapy clinical trials with AAV vectors are showing promising outcomes for hemophilia, but the existence of antibodies against AAV limits the in vivo gene therapy application. As an alternate, ex vivo HSPC gene-editing approach, where α-globin targeted integration of FIX-R338L transgene, was developed to express FIX in erythroid cells [26]. This indicates that HDR gene editing of HSPCs could also be used as a delivery system for therapeutic proteins.

Pyruvate kinase deficiency (PKD) disease: Mutations in the pyruvate kinase Isozymes R/L (PKLR) gene cause premature destruction of red blood cells resulting in PKD disease. Insertion of codon-optimized pyruvate kinase cDNA at its endogenous locus and puromycin selection of targeted cells to achieve 70% of gene-corrected cells is an exciting approach. However, poor in vivo engraftment of in vitro selected cells (< 1% HDR cells) remains a big obstacle to this approach [27].

### Primary immunodeficiency disorders

X-Linked chronic granulomatous disease (X-CGD): Mutations in the Cytochrome B-245 Beta Chain (CYBB) gene results in defective production of antimicrobial reactive oxygen species. CYBB gene mutation has been corrected with ZFNs coupled with AAV6 donors in CGD patient-derived HSPCs achieving 58% HDR in vitro and 6–16% in vivo [28]. CRISPR/Cas9 RNP and ssODN-mediated correction of C676T point mutation with an in vitro efficiency of 20% and in vivo efficiency of 13% was achieved [29].

X-linked severe combined immunodeficiency (SCID-X1): Mutations in γ chain (γc) encoding interleukin 2 receptor subunit gamma (IL2RG) gene, a major subunit of encoding for common γ chain (γc), leads to SCID-X1 [30]. Endogenous insertion of IL2RG cDNA using ZFN with IDLV or AAV6 was achieved with an efficiency of 10% & 25% in wild-type and patient HSPCs, respectively [31]. Similarly, with CRISPR-Cas9 and AAV6 donors, the insertion frequency was achieved up to 45% in patient HSPCs in vitro and functional correction was achieved in 10–20% of LT-HSCs in vivo [30]*.*

Wiskott–Aldrich syndrome (WAS): Mutations in WAS gene result in reduced levels of WAS protein. CRISPR/Cas9-mediated insertion of WAS cDNA into the endogenous site was achieved up to 47.9% in patient HSPCs resulting in restoration of WASp expression in 49% of the cells. *In vivo* studies showed the targeted insertion frequency of 40.7% and 37% in hCD45 + cells & hCD19 + B-cells, respectively [17].

Immune dysregulation, polyendocrinopathy, enteropathy, X-linked (IPEX) syndrome: IPEX syndrome occurs due to the mutations in the fox head box protein 3 (FOXP3) gene, a critical transcription factor necessary for regulatory T-cells (Tregs). Using the CRISPR-Cas9/AAV6-mediated gene editing, FOXP3 cDNA was inserted into its endogenous locus and FOXP3 expression was restored in 29 ± 8% of edited HSPCs that are capable of partially reconstituting Tregs (CD4^+^ CD25^+^ FOXP3^+^) with retention of 60% edited cells in vivo [32].

### Metabolic disorders

Mucopolysaccharidosis type I: Mutations in alpha-L-iduronidase (IDUA) gene reduce the expression of IDUA causing glycosaminoglycan accumulation in lysosomes resulting the diseases [33, 34]. Targeted insertion of IDUA gene to the CCR5 safe harbor locus using AAV6 donors resulted in higher HDR frequencies up to 54% ± 10 and 44% ± 7 in CB and PB HSPCs, respectively, and a fivefold decrease in long-term engraftment of gene-manipulated cells [34].

Gaucher disease: Mutations in glucocerebrosidase gene results in insufficient levels of glucocerebrosidase (GCase). In HSPCs, targeted insertion of glucocerebrosidase cassettes to the human CCR5 safe harbor locus using AAV6 donor templates resulted in HDR frequencies up to 51.5 ± 9.1% [33]. This monocyte-/macrophage-specific expression strategy generated supraphysiologic GCase in vivo*.*

## Challenges with HDR gene editing in HSPCs

Homology-directed repair holds a great potential in gene therapy as it allows precise customization of the genome, but this technique is not exempt from limitations. Although the gene-editing techniques have become well established through the years, HDR-based manipulation strategies, especially with HSCs, need rigorous improvization for successful clinical translation.

### Design and delivery of donor DNA template

Unlike the naturally occurring homologous recombination where sister chromatids act as the correction template, HDR-mediated gene-editing experiments require exogenous donor DNA template. The donor template can be customized in a versatile manner for the incorporation of DNA modifications of single nucleotide to several kilobases. However, a strong synergy of the RNPs and ssODNs is required for efficient editing and the addition of donor template can possibly hinder the delivery potential. Importantly, the donor template requires extensive characterization of the length of the homologous arms, polarity, PAM shield and the backbone modifications for an efficient HDR [15].

### Toxicity of exogenous oligonucleotide templates

A major challenge while using oligo donor is elicitation of immune response in the target cells. Oligo donors could mimic pathogen entry and the host cell, incapable of distinguishing both, activates Interferon gamma (IFNγ) mediated inflammatory immune response leading to apoptosis. In addition, free/exposed DNA ends in ssODNs are prone to exonuclease attack, thereby reducing the availability of donor templates for HDR. While the capped DNA ends in AAV plasmids confer better stability, their larger size increases the nucleotide burden [35, 36] Gene-editing-mediated cellular toxicity is also triggered by p53 pathway, as they are the immediate responders to the DNA damage, leading to apoptosis and gradual diminishment of cells. The p53 activity is increased by the donor on the introduction of DNA templates generating more cytotoxicity than editing with RNP [37].

### Cell cycle restrictions

HDR editing in HSPCs is challenging as the templated repair is permissive only during the S/G2 phases of the cell cycle. Hence, HSPCs and in particularly primitive HSCs, exhibit low susceptibility to HDR editing. While cytokine stimulation and prolonged culture are used to push HSPCs into cycling, these conditions are also associated with impairment in the engraftment of the HSPCs. The conventional cell cycle synchronization strategies are also observed to be challenging in HSPCs as there are no reports on the engraftment potential of synchronized HSPCs [38].

### Poor HDR editing efficiency in vitro

The overall efficiency of HDR gene editing remains low than the non-template correction mechanisms-NHEJ & MMEJ. While greater than 90% NHEJ gene-editing efficiency can be achieved, there are no reports on HDR gene-editing efficiency of > 50%. While selection of HDR positive cells can increase the frequency of HDR-edited cells, such a selection approach may not be possible with ssODN-based gene editing. Additionally, HDR-mediated large insertions occur at much lower rates than single nucleotide changes and small insertions. Also, different loci exhibit variabilities in the frequency of editing events due chromosome status and inherent bias toward NHEJ/MMEJ [39, 40].

### Reduced engraftment and long-term repopulating capacity in vivo

While < 50% of HSPCs are undergoing HDR gene editing in vitro, the xeno-transplantation experiments have indicated an additional problem of reduction in the frequency of HDR edited cells in vivo. HDR editing showed disappointing results in transplantation assays, with dramatic reduction in the engraftment of HDR cells. This may be due to one of the following factors—HDR levels are higher in the progenitor population than in primitive HSCs/long-term repopulating cells resistant to the templated DNA repair/the cells that have undergone HDR lose stemness and are not retained in vivo. Thus, there is always a fundamental imbalance between HDR editing and the stemness of the HSPCs [30, 40–42].

### Scar in gene locus

The HSPCs that were edited for HDR will contain a heterogenous pool of edited cells either homozygous, heterozygous or InDel only. In Some scenarios, the cells with InDels may be of concern. ssODN-mediated correction of SCD mutation in HBB locus results in correction of disease-causing mutation, but a fraction of cells with InDels can disrupt β-globin expression resulting in β-thalassemia like phenotype. Such unintended editing may not be a concern in many diseases. However, a precise edit is desirable [43].

## Strategies to overcome the limitations in HDR-based gene manipulation

Despite the limitations and difficulties in HDR-mediated gene editing, their potency in targeted editing is promising. So, over the years, several strategies are explored for improving HDR editing.

### Inhibition of competitive NHEJ pathway

Inhibition of proteins involved in different steps of the NHEJ repair pathway is a major approach to increase HDR efficiency. While stable knock-down of key repair proteins produce detrimental effects, usage of small molecules provides better solution of transient inhibition. SCR-7, NU7441, NU7026, STL127685, IC86621, M3814 and KU-0060648 are some of the small molecules tested to inhibit NHEJ and enhance the HDR. SCR-7 binds to the DNA-binding domain of ligase IV to inhibit NHEJ and improves HDR frequency in mammalian cell lines. Treatment with SCR7 resulted in an additive effect when combined with Adenovirus 4 (Ad4) E1B55K and E4orf6 proteins and improved the insertions of large transgene into Kell locus. NU7026, a DNA-dependent protein kinase (DNA-PK) inhibitor, improves HDR in iPSCs. Several other DNA-PK inhibitors like NU-7441 and KU-0060648 increased plasmid and ssODN-mediated HDR in HEK 293 T cells. NU-7441 increased the AAV6 donor-mediated HDR efficiency in iPSCs. IC86621 and M3814 enhance the HDR in V3 cells and k562 cells, respectively. CRISPY Mix (a mixture of small molecules: trichostatin A, MLN4924, NU7026 and NSC 15520) is shown to increase the HDR in iPSCs [5–7, 19]. Since most of the molecules for NHEJ inhibition have been tested in different human cell types but not in HSPCs, the above-mentioned molecules can be tested in HSPCs to enhance the HDR.

### Activation of HDR promoting factors

L755507, resveratrol, brefeldin A, RS-1, MLN4924 and NSC 15520 are the few molecules tested for enhancing HDR. Both L755507 and resveratrol increased HDR efficiency in porcine fetal fibroblast by increasing the expression of the key HDR factors. In addition, L755507 and brefeldin promote insertion of large fragment into ACTA2 locus in mouse embryonic stem cells (mESCs). Farrerol, a herbal compound, is found to enhance the HDR rates in HEK293FT cells and mESCs [44]. RS-1 enhances HDR in HEK-293A and U2OS osteosarcoma cell lines by recruiting RAD51. MLN4924 increases the extent of DNA end resection at double-stranded break site and promotes HDR in iPSCs. NSC 15520 which blocks the interaction of RPA to RAD9 and to p53 increases the HDR in iPSCs [5, 6, 19]. Though several NHEJ-independent factors are employed to increase HDR, efficiency of the small molecules varies based on the cell types and gene locus. The functional repercussions of small molecule-treated gene-edited HSPCs need careful validation since it is highly unexplored. Concentration of small molecules and experimental conditions should be further optimized in order to achieve high HDR efficiency in HSPCs.

### Altering the chromatin state

Chromatin state is shown to influence HDR gene editing and altering the chromatin state through modulation of HDAC activity, and histone octamer complex around the DNA affects the donor DNA integration. Entinostat (HDAC1/2/3 inhibitor) and panobinostat (Pan HDAC inhibitor) increase the HDR rate in HEK cell lines [45]. TSA, a HDAC 1/2 inhibitor, increases the frequency of HDR by 40% when used with RNP and AAV6 donor [46]. Valproic acid (VPA), a Class I (HDAC1,2,3,8) and IIa (HDAC4,5,7,9) HDAC inhibitor, enhances the HDR in human embryonic stem cells [47]. Similarly, romidepsin (Class I, IIa, IIb (HDAC6,10) & IV (HDAC11) and VPA increase the gene targeting efficiency in mESCs [48]. All these suggest that HDAC inhibition facilitates the Cas9 access to target DNA and increases the gene-editing frequencies, thereby enhancing the HDR efficiency.

### Altering the HSPC cell cycle

Cell cycle status influences the HDR outcomes, and one potential option is to increase the proportion of cells in S and G2 phases of the cell cycle. Restricting gene editing to S/G2 phase by Cas9-geminin fusion allowed transient Cas9 activity in S/G2 phase, thereby increasing the HDR/NHEJ ratio. Simultaneous treatment of hGemCas9 and a Cyclin-dependent kinase 1 inhibitor RO-3306 showed an increase in HDR levels in HSPCs. XL-413, an inhibitor of Cycle 7-related protein kinase (CDC7), delayed the cells in S phase and increases the HDR. HSPCs were also allowed for a controlled cycling to increase the HDR by 5- to 6-fold, and then, quiescence was introduced with Rapamycin and CHIR99021. Nocodazole, a microtubule polymerization inhibitor, and aphidicolin, a G1/S phase inhibitor, increased the HDR rates in HEK 293 cells. Microtubule polymerization inhibitor ABT-751 increased the gene integration in HSPCs [5, 19]. Although different cell synchronizing strategies have been used in HSPCs, the effect of synchronization on the stemness, genetic stability and differentiation potential of stem cells needs further investigation.

### Lowering donor template toxicity

Cell toxicity varies with the types of donor template used with the dsDNA, exhibiting higher toxicity than ssDNA oligos. Chemical modifications such as phosphorothiate (PS) modifications at the 3′ and 5′ end of the ssODN lower exonuclease attack, thus reducing cellular toxicity [35]. Since the naked DNA template is potentially toxic to the cells, use of chromatin DNA template which appears to be natural form of DNA lowers the template-associated toxicity [49]. Nuclease-induced DSB leads to the activation of p53 pathway thereby influences the survival of cells. Ameliorating the p53 mediated effects with the help of a dominant negative p53 protein helps to lower the donor template toxicity and improve the viability of edited HSPCs [37].

### Increasing donor availability at the target site

**Fig. 2:**
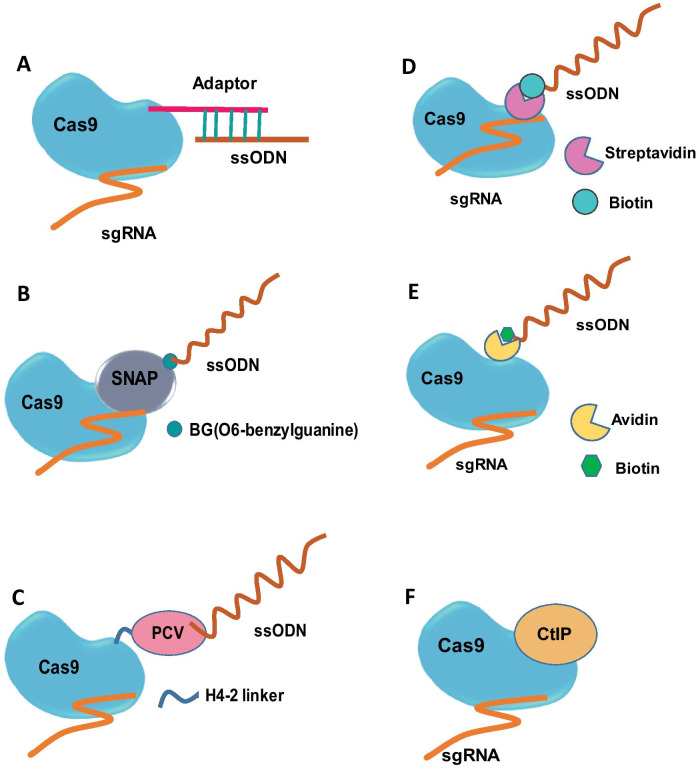
Strategies for increasing the donor availability at Cas9 cut site. **a** Thiol-maleimide chemistry-based Cas9 and ssODN conjugation. A short oligonucleotide adaptor is attached to the Cas9 via thiol-maleimide and appended with ssODN by base pairing. **b** Cas9-ssODN linking using snap-tag technology. Snap-tag fused Cas9 is covalently linked to the BG coupled ssODN. **c** RNP-ssODN tethering using HUH endonuclease. Cas9 is fused to the HUH endonuclease PCV Rep protein via H4-2 linker. Then, ssODN is covalently attached to the HUH endonuclease PCV. **d** In S1mplex system, sgRNA modified using streptavidin-binding aptamer is linked to the biotinylated ssODN, forming RNP-ssODN complex. **e** Cas9 is fused to the avidin via flexible linker and Cas9 is linked to biotin modified ssODN. **f** Direct fusion of CtIP and Cas9 nuclease

Increase in global donor concentration is shown to have a direct impact on the HDR events but affects the viability of HSPCs. As an alternative, the donor DNA can be concentrated at the Cas9 cleavage sites by physical linking of the ssODN to the Cas9 using various linkage chemistry. Cas9-ssODN conjugation by thiol-maleimide chemistry increased the HDR in INS-1E cells (Fig. 2a). Cas9 fusion proteins linked to the O^6^-benylguanine coupled ssODN (Fig. 2b) increased the precise correction rates in HEK 293 T cells. ssODN covalently tethered to the Cas9-porcine circovirus 2 rep protein via fused HUH endonuclease increased the HDR in HEK293T (Fig. 2c). In S1mplex system, biotinylated ssODN donor linked to the gRNA, modified with streptavidin–aptamer complex, increased the HDR/indel ratio in HSPCs (Fig. 2d). Similarly, biotin modified ssODN is linked to the avidin fused Cas9 via flexible linker (Fig. 2e) achieving higher HDR frequency in mouse embryos (Fig. 2e). Testing these approaches in HSPCs could provide interesting results [5, 50].

### Global expression of HDR promoting factors

HDR-enhancing factors have been extensively tested for application in gene editing, and different factors such as RAD51, RAD52, RAD54 and BRCA1 were overexpressed globally for HDR elevation. Coexpression of RAD51/RAD54 is shown to increase the HDR efficiency in HT-1080 cell lines. Similarly, the expression of either wild type or hyper-recombination variants of BRCA1 increased the HDR efficiency in HEK-293A cell lines. RAD52 motif protein 1 (RDM1) prevents the G2/M cell cycle arrest and thereby enhances the HDR frequency, and Cas9-RDM1 fusion variant is yet to be tested in human cells including HSPCs. Inhibition of 53BP1 by an ubiquitin variant (i53) enhanced the HDR efficiency with both AAV6 and ssODN donor. Co-expression of both factors, dn53BP1 and RAD 52, increased the frequency of HDR at multiple loci in HEK 293 cells and iPSCs. Overall, this technology can be applied in HSPCs to improve HDR and achieve higher rates of gene correction. Though several strategies are employed to achieve global expression of HDR promoting factors, further improvements must be necessary to achieve successful HDR-based gene therapy in HSPCs [4, 51, 52].

## Conclusion

The availability of innovative tools presents the scope for next-generation therapeutics for the treatment of genetic disorders. The rapid evolution of gene-editing tools and cutting-edge CRISPR/Cas9 technology has allowed development of more efficient and feasible gene therapy approaches than viral vectors. Reports from NHEJ mediated gene-editing approach showed promising results in the on-going clinical trials. HDR gene editing has not yet reached the clinical studies owing to the low efficiency and the complexities involved. The recent technologies such as base editing and prime editing provide a strong challenge to HDR editing as their efficiency is observed to be superior to HDR gene editing. However, the IND approvals for UCSF/UCLA approach of Cas9-ssODN-mediated SCD correction and Standford university approach of Cas9-AAV6-mediated SCD correction provide lots of hope for HDR gene editing. With the success of HDR-based gene editing, we are expected to witness the success of targeted gene therapy for several genetic disorders in future. Alongside the technical research and advancement for gene therapy, it is also important to design a cost-effective treatment module so that the therapy is accessible to most patients, especially in developing countries.

## Data Availability

Not applicable.

## Abbreviations

AAVs: Adeno-associated vectors
AdVs: Adenovirus vectors
BIR: Break-induced replication
BRCA1: Breast cancer type 1
cDNA: Complementary DNA
CRISPR-Cas9: Clustered regularlyy interspaced short palindromic repeats-associated RNA-guided Cas9
DNA-PKcs: DNA-dependent protein kinase catalytic subunit
DSB: Double-stranded break
FOXP3: Fox head box protein 3
HAT: Histone acetyltransferase
HBB: Hemoglobin subunit beta
HbS: Hemoglobin S
HDAC: Histone deacetylase
HDR: Homology-directed repair
HSPCs: Hematopoietic stem and progenitor cells
IDLVs: Integrase defective lentiviral vectors
IPEX: Immune dysregulation, polyendocrinopathy, enteropathy, X-linked
iPSCs: Induced pluripotent stem cells
MMEJ: Microhomology-mediated end joining
NADPH: Nicotinamide adenine dinucleotide phosphate oxidase 2 (NOX2)
NHEJ: Non-homologous end joining
SDSA: Synthesis-dependent stranded annealing
SSA: Single-stranded annealing
ssODNs: Single-stranded deoxynucleotides
SSTR: Synthesis-dependent stranded annealing
TALENs: Transcription activator-like nucleases
ZFNs: Zinc finger nucleases
